# Genome Sequence of the Multiantibiotic-Resistant *Enterococcus faecium* Strain C68 and Insights on the pLRM23 Colonization Plasmid

**DOI:** 10.1128/genomeA.01719-15

**Published:** 2016-05-05

**Authors:** Mónica García-Solache, Louis B. Rice

**Affiliations:** Department of Medicine, Rhode Island Hospital, Warren Alpert Medical School of Brown University, Providence, Rhode Island, USA

## Abstract

*Enterococcus faecium* infections are a rising concern in hospital settings. Vancomycin-resistant enterococci colonize the gastrointestinal tract and replace nonresistant strains, complicating the treatment of debilitated patients. Here, we present a polished genome of the multiantibiotic-resistant strain C68, which was obtained as a clinical isolate and is a useful experimental strain.

## GENOME ANNOUNCEMENT

*Enterococcus faecium* is one of the most insidious nosocomial pathogens, largely due to high intrinsic levels of resistance to antibiotics and a remarkable genome plasticity that favors acquisition of *de novo* resistance (1). Here, we report the polished genome sequence of clinical isolate and commonly used laboratory strain C68. Using PacBio sequencing, we were able to determine that the C68 chromosome is 2.875 Mb, with a 38.7% G+C content and 2,685 protein-coding genes. Additionally, we recovered a single 217-kb contig corresponding to the pLRM23 plasmid, which has been implicated in gastrointestinal colonization and cotransfer of antibiotic resistance genes (2–4). pLRM23 contains 208 protein-coding genes, including one replicase (*repA*), a putative conjugation gene (*traE)*, and a resolvase gene, 49 hypothetical proteins, and 56 genes related to insertion elements; also, it is enriched in phosphoenolpyruvate:carbohydrate phosphotransferase system (PTS) genes, including the PTS-mannose operon, which was previously associated with gastrointestinal colonization (5). The plasmid does not contain antibiotic resistance genes or other additional virulence factors. The pLRM23 G+C content is 35.6%. We also identified three other putative plasmids of 78, 45, and 15 kb.

DNA from *E. faecium* C68 was extracted using Qiagen Genomic tip-100 (Qiagen, Valencia, CA). Pacific Biosciences RSII single-molecule real-time (SMRT) sequencing (Pacific Biosciences, Menlo Park, CA) was performed, using a large-insert library (~10-kb insert) with a single PacBio RSII cell. Genome assembly was done using the Hierarchical Genome Assembly Process (HGAP) version 3, with a minimum read length set to 5,000 kb and 4% error rate allowed. The assembly presented here has annotation added by the NCBI Prokaryotic Genome Annotation Pipeline.

### Nucleotide sequence accession numbers.

This whole-genome shotgun project has been deposited at DDBJ/ENA/GenBank under the accession no. LPUE00000000. The version described in this paper is version LPUE01000000.
